# Dinucleotide composition representation -based deep learning to predict scoliosis-associated Fibrillin-1 genotypes

**DOI:** 10.3389/fgene.2024.1492226

**Published:** 2024-10-22

**Authors:** Sen Zhang, Li-Na Dai, Qi Yin, Xiao-Ping Kang, Dan-Dan Zeng, Tao Jiang, Guang-Yu Zhao, Xiao-He Li, Jing Li

**Affiliations:** ^1^ State Key Laboratory of Pathogen and Biosecurity, Academy of Military Medical Sciences, Beijing, China; ^2^ College of Basic Medical Sciences, Inner Mongolia Medical University, Hohhot, China; ^3^ Laboratory of Advanced Biotechnology, Academy of Military Medical Sciences, Beijing, China; ^4^ College of Veterinary Medicine, Shanxi Agricultural University, Jinzhong, China

**Keywords:** scoliosis, genotypes, deep learning, FBN1, genome composition

## Abstract

**Introduction:**

Scoliosis is a pathological spine structure deformation, predominantly classified as “idiopathic” due to its unknown etiology. However, it has been suggested that scoliosis may be linked to polygenic backgrounds. It is crucial to identify potential Adolescent Idiopathic Scoliosis (AIS)-related genetic backgrounds before scoliosis onset.

**Methods:**

The present study was designed to intelligently parse, decompose and predict AIS-related variants in ClinVar database. Possible AIS-related variant records downloaded from ClinVar were parsed for various labels, decomposed for Dinucleotide Compositional Representation (DCR) and other traits, screened for high-risk genes with statistical analysis, and then learned intelligently with deep learning to predict high-risk AIS genotypes.

**Results:**

Results demonstrated that the present framework is composed of all technical sections of data parsing, scoliosis genotyping, genome encoding, machine learning (ML)/deep learning (DL) and scoliosis genotype predicting. 58,000 scoliosis-related records were automatically parsed and statistically analyzed for high-risk genes and genotypes, such as *FBN1*, *LAMA2* and *SPG11*. All variant genes were decomposed for DCR and other traits. Unsupervised ML indicated marked inter-group separation and intra-group clustering of the DCR of *FBN1*, *LAMA2* or *SPG11* for the five types of variants (Pathogenic, Pathogeniclikely, Benign, Benignlikely and Uncertain). A FBN1 DCR-based Convolutional Neural Network (CNN) was trained for Pathogenic and Benign/ Benignlikely variants performed accurately on validation data and predicted 179 high-risk scoliosis variants. The trained predictor was interpretable for the similar distribution of variant types and variant locations within 2D structure units in the predicted 3D structure of *FBN1*.

**Discussion:**

In summary, scoliosis risk is predictable by deep learning based on genomic decomposed features of DCR. DCR-based classifier has predicted more scoliosis risk *FBN1* variants in ClinVar database. DCR-based models would be promising for genotype-to-phenotype prediction for more disease types.

## 1 Introduction

Scoliosis is a three-dimensional structural spine deformation, characterized by a lateral deviation of at least 10° with a rotation of the vertebra and usually associated with a reduction of normal kyphotic curvature of the spine (Choudhry et al., 2016). As much as 80% of all scoliosis is termed “idiopathic” or of unknown etiology. AIS is diagnosed when the deformity Cobb angle goes above 10° in children and adolescents after the age of 10 and until skeletal maturity (Perez-Machado et al., 2020). The prevalence of AIS ranged from 2% to 5.2% in various countries (Cilli et al., 2009; Konieczny et al., 2013; Soucacos et al., 1997; Wong et al., 2005), commonly with a female/male ratio of 1.5:1 to 3:1, respectively (Konieczny et al., 2013), and with a 90% presentation of right-sided thoracic curve (Cheng et al., 2015). Numerous hypotheses have been proposed regarding the effects of a variety of biomedical abnormalities on AIS, including neurologic development, spinal growth, bone metabolism, metabolic pathways, endocrine factors, and sex hormones (Kulis et al., 2015; Raczkowski, 2007). However, AIS cannot be attributed to clear causes for 80% of cases, but it has been suggested that it may be genetically related, to a polygenic background or to a quantitative trait locus, which may vary from several different genetic loci (Kikanloo et al., 2019). One fourth of AIS patients have a relative with the condition, but the inheritance pattern is variable (Choudhry et al., 2016). Considering the significant physical and psychological suffering and the economic burden of medical intervention post AIS onset, it is crucial to identify and predict potential AIS-related genetic and epigenetic variants before the scoliosis onset.

Emerging evidence indicates a correlation between genomic variation and the risk of AIS, facilitated by the analysis of genomic sequencing data obtained through next-generation sequencing (NGS) and third-generation sequencing technologies. Bibliometric analysis based on the reports about AIS risk found that high frequent polymorphism in fibrillin (*FBN*) gene, estrogen receptor gene, calmodulin, collagen gene and Ladybird Homeobox 1 (*LBX1*) might involve in physiological or (and) pathological processes, such as menarche, Bone formation, disc degeneration, melatonin signaling dysfunction, and cerebrospinal fluid flow in AIS (Jiang S. et al., 2023). And most of these research was analyzed by traditional link analysis (Duance et al., 1998) or more popular tool of Genome-wide association studies (GWAS) (Kou et al., 2013; Ogura et al., 2015; Sharma et al., 2011; 2015; Takahashi et al., 2011; Ushiki et al., 2024). A GWAS comprising 79,211 subjects revealed a fine-tune deregulation of Cobb angle by 187,633 Single Nucleotide Polymorphisms (SNP)s in multiple genes, with a r^2^ of 0.7 (Otomo et al., 2021). More GWAS studies identified AIS-associated genes, like *FBN1* (Buchan et al., 2014; Sheng et al., 2019), *LBX1* (Takahashi et al., 2011), G protein-coupled receptor (*GPR126*) (Kou et al., 2013), adherents junction associated protein 1 (*AJAP1*) (Zhu et al., 2015), basonuclin 2(*BNC2*) (Ogura et al., 2015), paired box 1 *(PAX1)* (Sharma et al., 2015) and so on. However, most of these cohort-based studies were limited to one or several specific population(s), lacking a landscaping view of AIS genetic backgrounds.

Disease variant prediction is based on the public archive of interpretations of clinically relevant variants (ClinVar) (Landrum et al., 2016) and the Human Gene Mutation Database (HGMD) (Stenson et al., 2003). ClinVar integrates and updates all freely available reported medically important variants and phenotypes, including scoliosis (Landrum et al., 2018; Landrum et al., 2014), and has been widely taken as a critical resource for advanced variant interpretation. And several studies based on the ClinVar resources have recognized more genetic variants possibly associated with vertebral malformations, such as a chromosome 1q22 microdeletion of *ASH1L* (Xi et al., 2020), series of SNPs in KIAA1217 (Al et al., 2020), and the 3′ UTR of *KLHL40* (Dofash et al., 2023). However, a comprehensive analysis of AIS-associated genetic variants is not available up to now. The complicated associations between genotypes and phenotypes are easier to identify with ML or DL approaches. A DL tool of AlphaMissense designed by DeepMind predicted accurately the effect of proteome-wide missense variant for various types of diseases (Cheng et al., 2023; Minton, 2023). More and more DL or ML tools predicted intelligently disease-associated phenotypes based on genotypes (Jo et al., 2023; Kotlarz et al., 2024). Similarly, our previously developed multiple tools performed well in predicting the adaptation phenotypes of viruses based on their genotypes in either coding region (Bei-Guang et al., 2022; Jiang X. et al., 2023; Li et al., 2023; Li et al., 2022) or UTR (Sun et al., 2014).

In the present study, we have analyzed the scoliosis-related genotypes in the ClinVar database with multiple ML approaches to screen top scoliosis-associated genes, and then built DL predictor for scoliosis-associated genotypes. The present study provided the most recent analysis on the scoliosis-related genetic variants in ClinVar database, and found several novel genes and genotypes which are associated to scoliosis.

## 2 Materials and methods

### 2.1 Preparation of scoliosis-related data and genome decomposing

Scoliosis-related variants and their annotations were downloaded from ClinVar database (https://www.ncbi.nlm.nih.gov/clinvar/); Data was cleaned to remove those variant samples with variant in intron, and was parsed for annotations, such as gene name, gene ID, variant type and others for each sample. The full coding DNA sequence (CDS) for each variant was generated based on the CDS of gene ID and its variant annotation, and variant CDS traits of dinuleotide (DNT), DCR, codon usage, codonpair and amino acid (AA) were decomposed with a reported decomposer (Li et al., 2022), respectively producing vectors with dimension of 48 (DNT), 1,536 (DCR), 64 (codon usage), 3,721 (codonpair) and 20 (AA) for each sample. The algorithm for counting DNT (Li et al., 2020), DCR (Li et al., 2022), codon, codonpair and AA (Jiang S. et al., 2023) were designed according to Formula 1‐5 respectively. Statistical description of variants was performed based on sample annotation information.

freqxnym=∑xnym∑i=116xnym,(x,y=T,C,A or G,m=n+1 for m≤3,m=n−2 for m=4,n=codon nt position 1,2,or 3 (1)

freqwixjykzl=∑wixjykzl∑i=1256wixjykzl,(w,x,y,z=T,C,A or G,j k=i+1 i+2,j  k=j k if j  k≤3,else j k=j−3  k−3,k=k+1,l=l if l≤3 else l=3,i=codon nt position 1,2,or 3 (2)

$$Freq\left(Codon\right)=count\left(Codon\right)\times 64\times \frac{3}{CDS\;length},codon=each\;of\;the\;64\;types\;of\;codons$$ (3)

$$Freq\left(codonpair\right)=count\left(codon\;pair\right)\times 3721\times \frac{3}{CDS\;length},codon\;pair=each\;pair\;of\;the\;64\;types\;of\;codons$$ (4)

$$Freq\left(AA\right)=count\left(AA\right)\times 20\times \frac{3}{CDS\;length},AA=each\;of\;the\;20\;types\;of\;amino\;acids$$ (5)

### 2.2 Unsupervised machine learning of the genomic composition of scoliosis-related genes

To learn the association of genome information with the scoliosis phenotype, in the gene of *FBN1*, *LAMA2* and *SPG11*, the clustering and separation of the variant samples were analyzed based on the DCR and other features of these genes. The composition feature vector of DNT, DCR, codon usage, codonpair and AA respectively with a dimension of 48, 1,536, 64, 3,721 and 20 was reduced to two main components with Uniform Manifold Approximation and Projection (UMAP) and were scattered with sample label of “Pathogenic”, “Pathogeniclikely”, “Benign”, “Benignlikely” and “Uncertain”. Hierarchical clustering of these samples was also performed with a python package of sns. clustermap, based on the Euclidean distance of the above-mentioned five types of features with each sample labelled. The components reduced from the compositional features were normalized with the following formula 6.

$$X_{normalized}=\left(X-X_{\min}\right)/\left(X_{\max}-X_{\min}\right)$$ (6)

### 2.3 Training of a Convolutional Neural Network (CNN) classifier for scoliosis genotypes

A CNN classifier was designed to predict scoliosis genotypes of *FBN1* based on genomic compositional features with the variants labelled as “Pathogenic” as positive samples and with the variants labelled as “Benign” or “Benignlikely” as negative samples. A random downsampling was performed to guarantee a sample balance between the two types of data. DCR with 1,536 dimensions were selected to train the classifier with a network structure of CNN. DCR data was then randomly split into training dataset and validation dataset with a 5-fold cross-validation method, then was reshaped into an array with the size of (6, 16, 16) and finally was input into the three-layer 3D-CNN model. The CNN models were set with a convolution kernel with size of (1, 3, 3), with a pooling layer of (1, 2, 2) via average pooling, and with a padding layer of (0, 1, 1), and with a stride of (1, 1, 1). The batch size, learning rate, and training epochs were optimized respectively. 768-dimensioned output from two rounds of convolution of the 1536-dimensioned DCR was linearly transformed for two times, firstly into 192-dimensioned and secondly into 2-dimensioned output, which was finally calculated with Softmax function (formula 7) to output the probability for each of the two scoliosis risks (positive and negative). Detailed parameters are epoch_num = 100, split_size = 0.2 (for training and validation dataset), lr = 0.005 and batch_size = 20. The classification performance of the models was evaluated by with receiver operating characteristic curve (ROC) and the area under curve (AUC), confusion matrix.

$$Softmax:f\left(x_{i}\right)=e^{x_{i}}/\sum_{j=1}^{J}e^{x_{j}}$$ (7)

### 2.4 Prediction and analysis of scoliosis genotypes

The variants labelled with “Pathogeniclikely” or “Uncertain”, from ClinVar database were assessed for their scoliosis risk with the trained CNN classifier based on their DCR features. The DCR of *FBN1* CDS was reshaped into a dimension of (6, 16, 16), and then were transformed into a tensor, then was input into the loaded model of “DCR-based 3D-CNN for scoliosis. txt”. The prediction of 1 for high scoliosis risk and 0 for low scoliosis risk and the probabilities for the two risk results were finally output. The scoliosis risk was further analyzed in more details by statistically describing distribution of these samples on various variant labels.

### 2.5 Structure prediction of truncated FBN1 with Alphafold2

To landscape the distribution of scoliosis variants on the 3D structure of FBN1, a reference FBN1 protein (NM_000138.5, NP_000129.3) with truncated N-terminal of 1,100 amino acids, where most of the amino acid variants were located, was utilized to predict its 3D structure. The structure prediction was performed with AlphaFold2 of offline version (Jumper et al., 2021). A virtual environment was first activated with the command “conda activate alphafold” under the same path as the FBN1 fasta file. Then the prediction was performed with the command “python/docker/run_ docker. py--fasta paths = Reference_NM_000138.5_FBN1. fasta--max_template_data = 2020-05–14”. The ranked0_.pdb was taken as the most optimized result and was visualized with PyMOL (version 2.5.7). All variants amino acids were manually labelled in red for the variants in the Pathogenic group, in purple for predicted risk variants in the Pathogeniclike group, and in violet for risk variants in the Uncertain group.

### 2.6 Statistics

Significance was evaluated with paired t-test for the Principal Component Analysis (PCA)-reduced PCA1 or PCA2 value of the full-connected layer between Pathogenic and Benign groups, and for the probability values of label 1 and label 0 either for negative or positive variants. GraphPad Prism (version 9.0.0) was utilized for statistical analysis and figure plotting. A *p*-value threshold of 0.05 was taken as statistical significance.

## 3 Results

### 3.1 Deep learning framework to predict scoliosis genotypes

**FIGURE 1:**
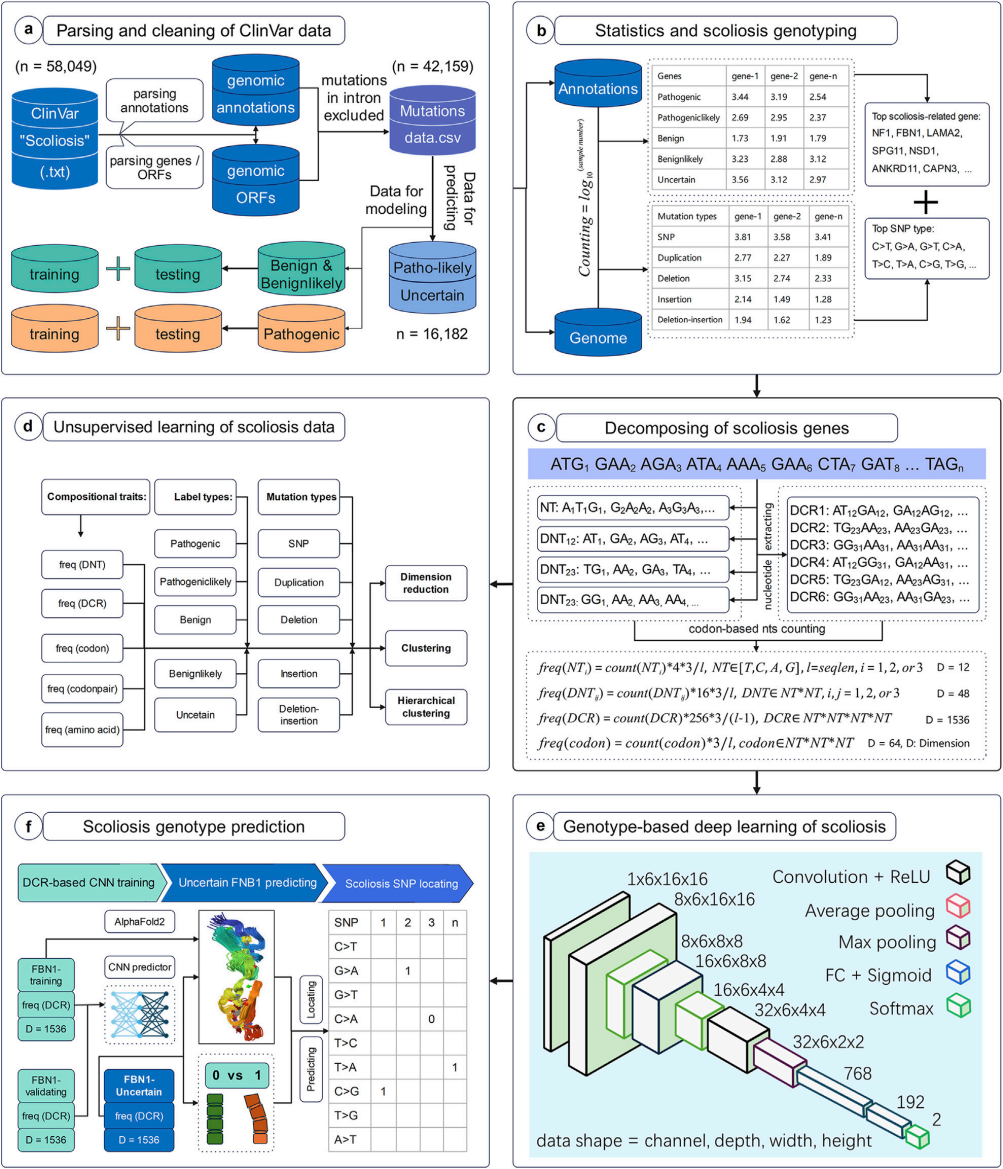
Workflow to decompose scoliosis-related genome and to predict scoliosis-related genotypes. The present workflow was designed for the parsing and cleaning of ClinVar data **(A)**, statistical analysis and screening for scoliosis-related genotypes **(B)**, decomposing scoliosis-related genes **(C)**, unsupervised learning analysis of compositional features of scoliosis-related genes **(D)**, deep learning network structure for predicting scoliosis genotypes **(E)**, and scoliosis genotype prediction **(F)**.

The workflow of this study was set to parse, decompose and predict the genotype with high scoliosis risk, with six successive models. Firstly, full data of variant samples associating with scoliosis or not were downloaded from NCBI and were parsed for their genotypes and annotations (Figure 1A). The distribution of these data on various types of annotation labels was statistically analyzed and the full CDS were generated based on their variant annotations (Figure 1B). Secondly, the genomic compositional traits, such as dinucleotides (DNTs), DCR, and others, for each scoliosis-related gene were calculated based on our previously reported algorithm (Figure 1C), and then were analyzed with unsupervised machine learning methods (Figure 1D). Finally, a Convolutional Neural Network (CNN) classifier based on DCR features of FBN1 was trained with two labels: high and low risk for scoliosis (Figure 1E). It was used to predict the scoliosis risk of variants with unclear risk and was interpreted by optimizing significant amino acids on the 3D structure of FBN1 (Figure 1F).

### 3.2 High frequent variations of *FBN1* and other genes associated with scoliosis

**FIGURE 2:**
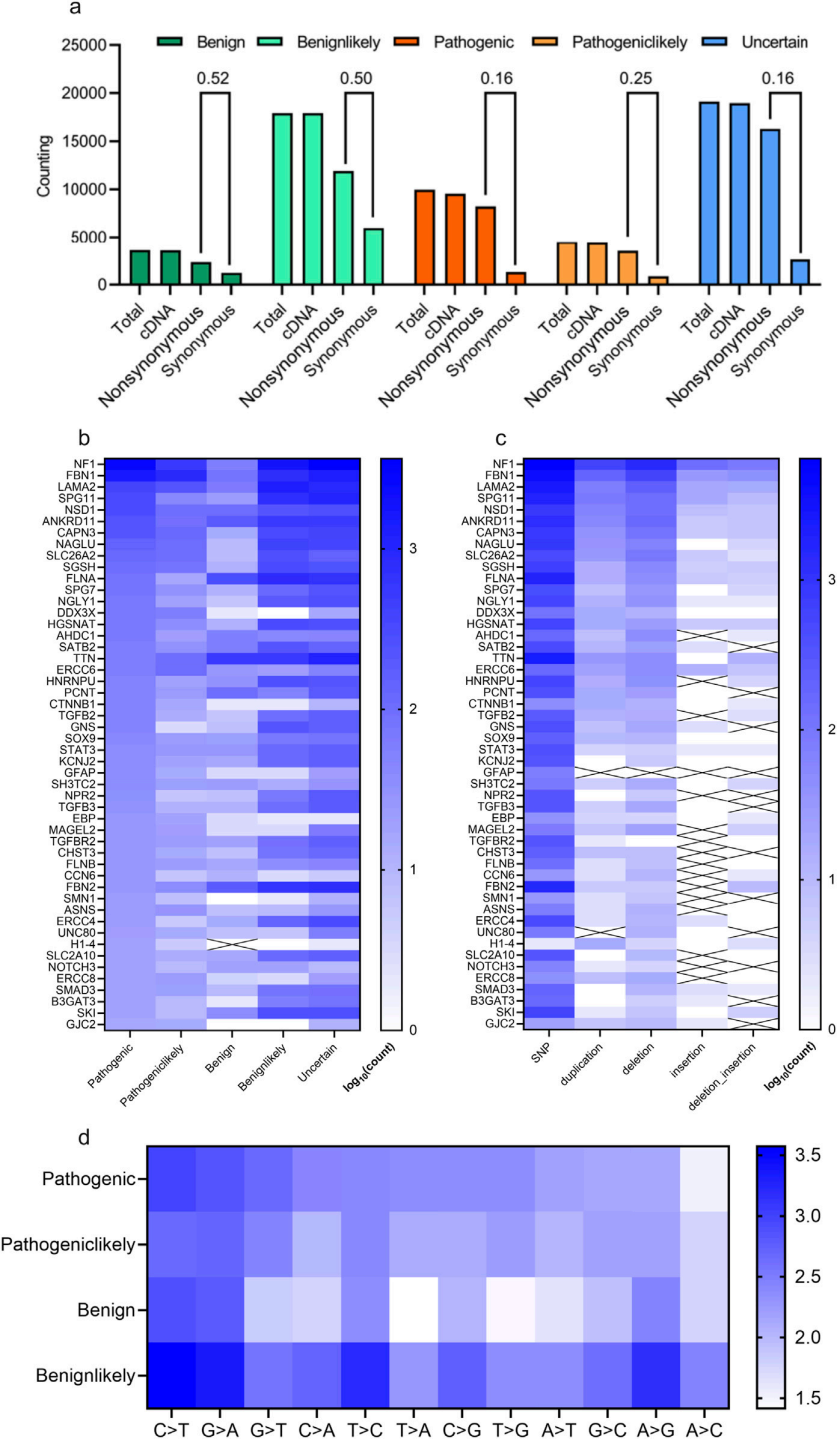
Statistical description of scoliosis-related genes and genomic mutations. **(A)** Counting and statistical analysis of the gene types (cDNA, nonsynonymous (could affecting protein), synonymous) for scoliosis risk types (Benign, Benignlikely, Pathogenic, Pathogeniclikely and Uncertain). **(B, C)** Heatmap of the counting results of genes for various scoliosis risk types **(B)** and for various mutation types **(C)**. **(D)** Heatmap of the counting various mutation types of scoliosis risk types (Benign, Benignlikely, Pathogenic and Pathogeniclikely).

The distribution of scoliosis-related variants was analyzed. Most of these variants whether with high scoliosis risk (Pathogenic or Pathogeniclikely), with low scoliosis risk (Benign or Benignlikely) or with unknown risk (Uncertain) are located within exon/cDNA, rather than within intron (almost similar value for Total and cDNA, Figure 2A). Most of the variants in cDNA were not synonymous, causing variants in protein level for scoliosis-pathogenic samples (Synonymous/Protein = 0.16 or 0.25 for the samples labelled with pathogenic or pathogeniclikely cDNA, Figure 2A). Counting of scoliosis-related genes indicated that *NF1, FBN1, LAMA2* and *SPG11* led the top list of genes concerned with scoliosis (Figure 2B). And single nucleotide polymorphism (SNP) dominated the variant type list for most scoliosis-related genes (Figure 2C). Most variant types for these SNPs were the base transition of C > T, G > A and G > T for pathogenic variants (Figure 2D).

### 3.3 DCR-based clustering and separation of *FBN1* and other genes for scoliosis

**FIGURE 3:**
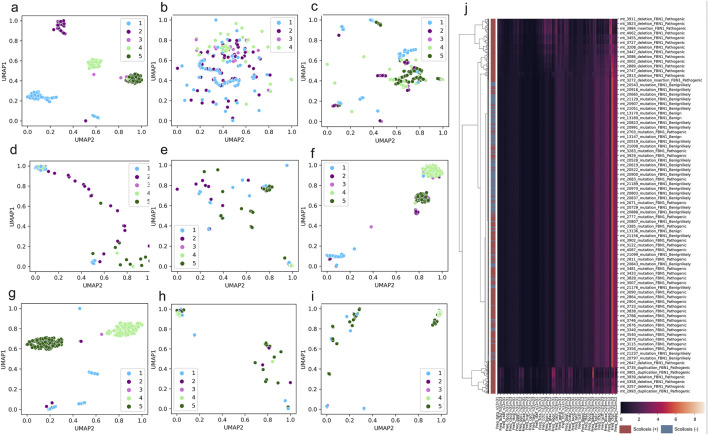
Significant clustering and separation of scoliosis-related genes and mutations on genomic DCR features. **(A–E)** Scatter plot of UMAP-reduced compositional features of DCR, DNT, codon, codonpair of randomly sampled *FBN1* variants. **(F, G)** Scatter plot of UMAP-reduced DCR of *LAMA2* and *SPG11*. **(H, I)** Scatter plot of UMAP-reduced AA of *LAMA2* and *SPG11*. Sample label types of Pathogenic, Pathogeniclikely, Benign, Benignlikely, and Uncertain were respectively annotated as 1-5. **(J)** Hierarchical of *FBN1* DCR features of the variants labelled with “Pathogenic” and “Pathogeniclikely” as red, “Benign” and “Benignlikely” as blue.

To overview the distribution of scoliosis-related variants based on genome decomposition traits, decomposed genome data of DCR and other traits was plotted with scoliosis risk labelled (high scoliosis risk: Pathogenic or Pathogeniclikely), low scoliosis risk: Benign or Benignlikely), post dimension reduction with Uniform Manifold Approximation and Projection (UMAP). A marked clustering of the samples with the same risk label and a long-distance separation of the samples with different labels were observed for DCR features of *FBN1* for the two main components of UMAP1 and UMAP2 (Figure 3A). Whereas these samples with the four labels were mixed in the distribution of UMAP1 and UMAP2, either for the trait of DNT (Figure 3B), codon (Figure 3C), codonpair (Figure 3D) or AA (Figure 3E) of *FBN1*. The intra-risk type clustering and inter-risk type separation were repeatedly observed on the DCR features of *LAMA2* and *SPG11* (Figures 3F, G), rather than on the AA features of the two genes (Figures 3H, I). The relatively hierarchical clustering (high scoliosis risk of Pathogenic vs. Benign and Benignlikely) was also indicated by DCR of *FBN1* (Figure 3J). However, such clustering was not significantly binary on the raw DCR data of *FBN1*, implying an incapability of the raw DCR data for a binary classification for the two scoliosis risk types and a need of feature optimization of DCR by deep learning.

### 3.4 Deep learning prediction of scoliosis genotypes of *FBN1*

**FIGURE 4:**
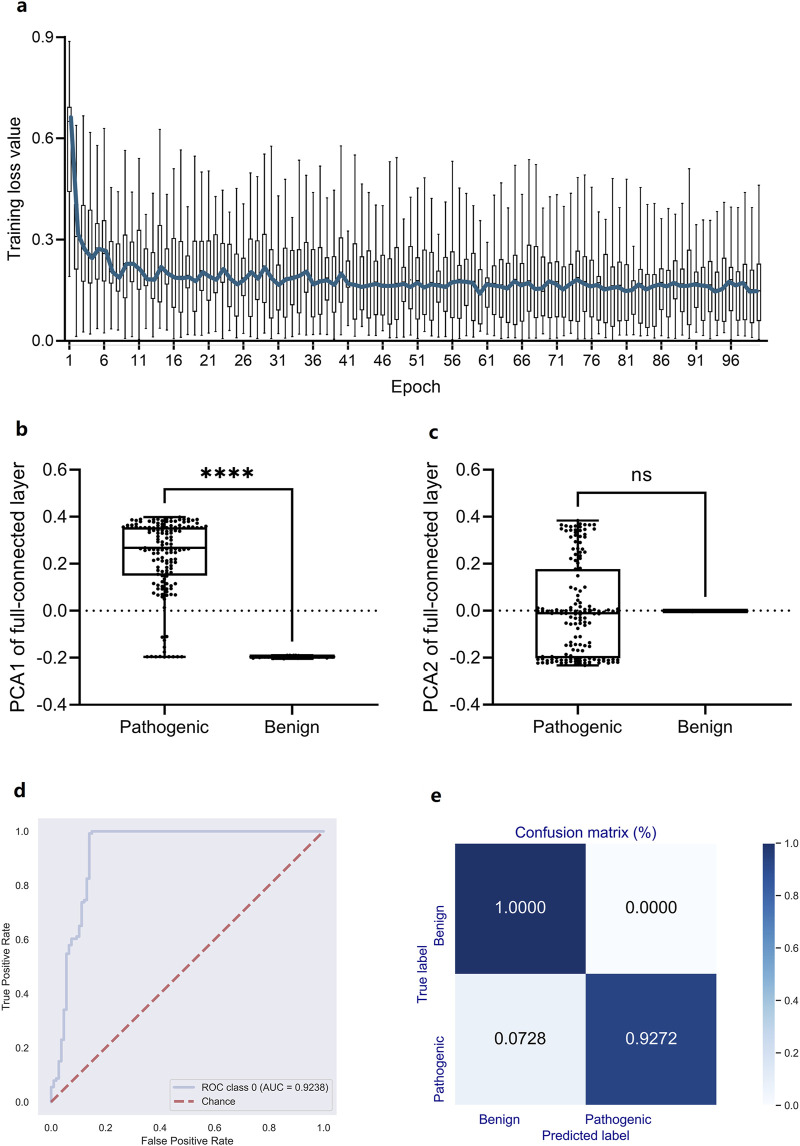
Performance of a DCR-based Convolutional Neural Network (CNN) classifier for predicting scoliosis-associated genotypes of Fibrillin-1 (FBN1). **(A)** Training loss per training epoch was plotted as a boxplot for all loss values per epoch, with the average value per epoch curved. **(B, C)** Comparison of the PCA1 **(B)** and PCA2 **(B)** reduced from the fully connected layer of the trained CNN classifier. ROC_AUC **(D)**, Confusion matrix **(E)** by the trained CNN classifier based on independently sampled variants. Benign: low scoliosis risk, Pathogenic: high scoliosis risk. ****p < 0.0001, ns: no significance.

In light of the high association between high-dimensional DCR features and scoliosis risk, however their nonlinear distribution depending on scoliosis type, a deep learning predictor based on the 1,536-dimension DCR trait was trained for scoliosis risk classification for the leading gene of scoliosis risk, *FBN1*. A CNN network (Li et al., 2023; Li et al., 2022) was utilized for the binary classification based on randomly sampled *FBN1* data. Firstly, a binary classifier of Convolutional Neural Networks (CNN) with labels of high risk (Pathogenic) and low risk (Benign or Benignlikely) was built based on DCR of *FBN1*. The 1536-dimension DCR were sequentially subject to two rounds of convolution, two times of linear transformation, and one-time Softmax transformation, and then outputted two classification labels of 1 (high) and 0 (low). The CNN classifier was quickly converged with optimized parameters, indicating an early decrease and following long micro-movement of training loss (Figure 4A). The fully connected layer of 768 dimensions was reduced with Principal Component Analysis (PCA) to visualize the concentration of key information of DCR by deep learning. It was indicated that the key difference between high and low risk data concentrated on the first component, showing a significant difference of PCA1 (*p* < 0.001, Figure 4B), whereas the insignificant difference of PCA2 (Figure 4C), between the two groups. Then the prediction performance was evaluated with an independent validation dataset with a confusion matrix and Receiver Operating Characteristic Curve_Area under Curve (ROC_AUC). A right angle-like ROC and an AUC value of more than 0.92 (Figure 4D) and a confusion matrix with 100% of accuracy for low risk data and 92.72% of accuracy for high risk data were obtained on independent training data (Figure 4E). Thus, DCR of *FBN1* was classifiable and predictable for high or low scoliosis risk, post convolutional transformation.

**FIGURE 5:**
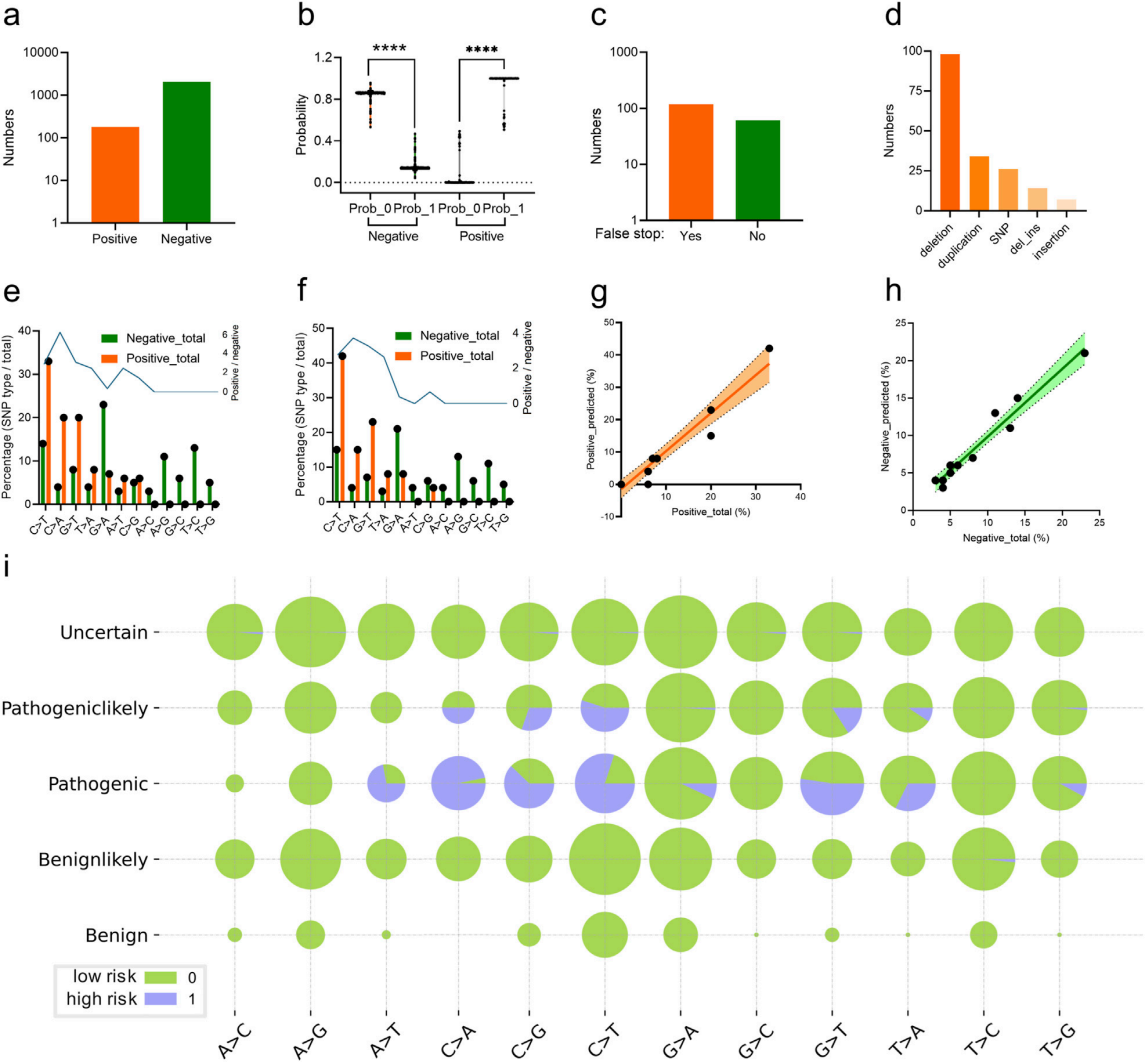
Analysis of CNN-predicted FBN1 variant samples with high scoliosis risk. Numbers **(A)** and probability **(B)** of the predicted *FBN1* variants with the high (positive) or low scoliosis risk (negative) for the variants, labelled with Pathogeniclikely or Uncertain, by the trained CNN classifier. The number of the predicted scoliosis-risk *FBN1* variants with (Yes) or without (No) false stop in coding region **(C)**, with mutation type of deletion, SNP, duplication, deletion_insertion or insertion **(D)**. Percentage of *FBN1* variants with various SNP mutation types, in both high risk (positive) or low risk (negative) for total scoliosis groups **(E)** or predicted scoliosis groups **(F)**. Correlation of the vector of percentage values for various SNP mutation types of high risk (positive) **(G)** or low risk (negative) **(H)** *FBN1* variants between total and predicted samples. The percentages of SNP mutation types in each of the five groups of variants were plotted **(I)**.

The trained CNN classifier was utilized to predict the scoliosis risk of the variant labelled with “Pathogeniclikely” or “Uncertain” from the ClinVar database. 179 variants (Supplementary Table S1) were predicted as high risk (label 1) and the other 2044 variants were low risk (label 0) based on their genomic DCR (indicated as positive and negative respectively, Figure 5A), with significant probability difference (*p* < 0.001 between label 0 and 1, respectively for both positive and negative groups, Figure 5B). There were 118 variants with false stop in *FBN1* CDS and 61 variants without false stop (Figure 5C). Deletion and duplication were the main variant types for the scoliosis risk variants (Figure 5D). In more detail, the frequency of various SNP types for all scoliosis risk variants and for predicted scoliosis risk variants were compared.

### 3.5 Interpretation of the CNN predictor: the scoliosis risk high frequency of N-terminal variation responsible for scoliosis

Interestingly, there were relatively high levels of SNP types of C > T, C > A, G > T and T > A were observed in either the Pathogenic group from the ClinVar database (Figure 5E) or the predicted scoliosis risk group (Figure 5F). Moreover, the frequency vector for these SNP types were highly similar between the two groups, indicating a linear correlation with high slope signification (Figure 5G). Such linear correlation was also observed for low scoliosis risk prediction in the frequency vector for these SNP types between the two groups (Figure 5H). Detailed percentages for high (label 1) and low (label 0) scoliosis risk variants for all five groups (Uncertain, Pathogeniclikely, Pathogenic, Benignlikely and Benign) were plotted (Figure 5I). Therefore, the DCR embedding of *FBN1* and the trained CNN classifier worked well in predicting scoliosis-risk variants, based on the interpretability of the high similarity in variant between pathogenic variants and predicted variants.

**FIGURE 6:**
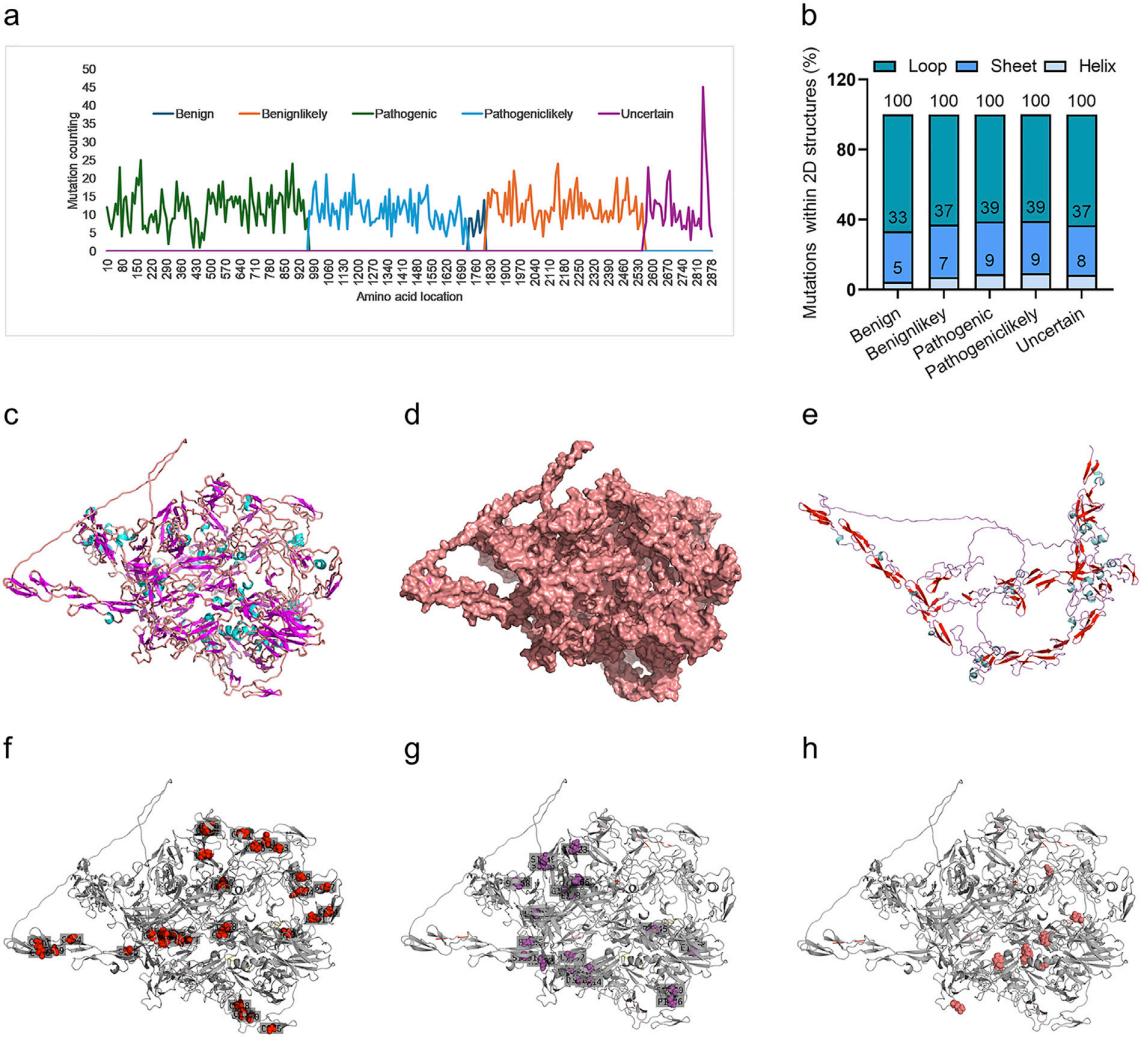
Distribution of scoliosis risk mutations in 1D sequence, 2D and 3D structures of FBN1 protein. Mutations within a sliding window of FBN1 protein sequence, with a sliding step of 10 AA, for Benign, Benignlikely, Pathogenic, Pathogeniclikely or Uncertain group **(A)**. Percentage of mutations in the 2D structure (Loop, Sheet, or Helix) of the alphafold2-predicted 3D structure of FBN1 **(B)**. The alphafold2-predicted 3D structure of full-length FBN1, showing AA form as a carton **(C)** or sphere **(D)**. The alphafold2-predicted 3D structure of the N-terminal (1-1100 AA) of FBN1, showing AA form as a carton **(E)**. The location with a mutation frequency of more than 5 times for Pathogenic **(F)**, Pathogeniclikely **(G)** or Uncertain **(H)** group.

We further interpreted the dependence of the predictor on protein sequence or structure. Firstly, the frequency of variants for all variants in each of the four groups was analyzed. It indicated a marked biased variant distribution on the *FBN1* sequence, with most of the pathogenic variants located in the N-terminal, followed by pathogeniclikely variant, with benign and uncertain variants in the C-terminal (Figure 6A). The variant distribution within the 2D structures of FBN1 was also analyzed. The 3D structure of the human FBN1 protein with Alphafold2 and mutated amino acid with the 2D structure of loop, helix and sheet was counted. It was shown that most of these variants were within the loop structure of FBN1, without marked differences among the four groups (Figure 6B). The full view of the 3D structure of full-length FBN1 was visualized in a cartoon (Figure 6C) and surface form (Figure 6D), indicating regular repeated units of beta-sheet and loop/alpha-helix. A truncated FBN1 with the 1,100 amino acids in the N-terminal indicated the repeated sheet-loop/helix units in more detail (Figure 6E). All the amino acid variants in FBN1 for the Pathogenic (Figure 6F), the Pathogeniclikely (Figure 6G) and the Uncertain group (Figure 6H) distributed uniformly, without any dependence on 2D structure. Taken together, the DCR-based CNN predictor for scoliosis-risk prediction was not dependent on the protein sequence or protein structure of FBN1.

## 4 Discussion

The exploring genome sequencing data by NGS and third generation sequencing poses a challenge to the widely utilized analysis tools for the association of genomic variation with disease risk. In response to the challenge, we presently designed an intelligent framework to explore the scoliosis-associated genes and their variants, based on all available genomic variant data about scoliosis. The framework was composed of all technical sections such as data parsing, scoliosis genotyping, genome encoding, machine/deep learning and scoliosis genotype predicting, implying a promising potential for digging the genotypes for any disease with potential genetics association. The data parsing section parsed all scoliosis associated data automatically and efficiently on a mobile workstation on more than 58, 000 records from ClinVar database, including various types of disease-related annotations. Moreover, the section was qualified to parse full-length gene sequences from the recorded variant annotation. Secondly, the high-risk genes and variants for scoliosis were easily screened based on statistical analysis by the genotyping section. Only the top second to fourth genes of *FBN1*, *LAMA2* and *SPG11* were analyzed in detail, given the consensus of the contribution of NF1 to an autosomal dominant disorder of Type I neurofibromatosis, which was usually complicated with scoliosis (Jett and Friedman, 2010), regardless of ranking first gene of *Neurofibromatosis 1* (*NF1*) for scoliosis high-risk genes. A high association of *FBN1* and the other two genes was observed with scoliosis.

Gene embedding is one of the key techniques for intelligent learning of disease-associated genes. Our reported genome embedding method, DCR was biologically interpretable in decomposing virus genes (Jiang X. et al., 2023; Li et al., 2023; Telenti et al., 2022; Zhang et al., 2024), and was competent in parsing the genes with varied sequence length. Thus, the three scoliosis-associated genes were decomposed with DCR, given the various sequence lengths of each of them, caused by varied variant types, such as insertion, deletion, duplication or deletion/insertion. Interestingly, the two reduced components of the 1536-dimensioned DCR features of either gene clustered within the same and separated among group(s) of Pathogenic, Pathogeniclikely, Benign, Benignlikely, and Uncertain variants, implying the potential of DCR to efficiently represent the genotype-phenotype association of scoliosis. In light of the representation significance of DCR, we built a DCR-based deep learning classifier to predict high-risk variants for scoliosis to assess the risk potential of the FBN1 variant labelled with Pathogeniclikely or Uncertain in the ClinVar database. A high prediction performance was observed for the classifier trained with randomly sampled samples of two groups of Pathogenic and Benign/Benignlikely FBN1 variants. The classifier predicted 179 scoliosis-associated variants which were labeled with Pathogeniclikely or Uncertain.

Since no gold standard for identifying the association of these predicted variants with scoliosis and no available tools to predict such association, more effort was paid to interpret or evaluate the reliability of the prediction by our deep learning classifier. Surprisingly, an extremely high similarity in variant types at cDNA level was observed in the scoliosis-associated variant difference between the group of recorded data (Pathogenic/Benign) and the group of predicted data (Positive/Negative), with an extreme correlation of variant types between the true and predicted variants. However, the high-frequent variant distributed differently in FBN1 protein sequence, between the group of recorded Pathogenic variants (N-terminal) and the group of predicted scoliosis risk variants (C-terminal). Interestingly, the predicted 3D structure of FBN1 indicated regular repeated units of beta-sheet and loop/alpha-helix in FBN1, and a similar distribution of mutated amino acids in the three 2D-structure types. Taking together the similar variant distribution at both mRNA and 2D-structure protein levels for recorded and predicted variants, the trained CNN classifier in the present study was reliable in predicting scoliosis risk genotypes, based on genomic DCR. In recent years, multiple machine/deep learning predictors have been built to predict various types of disease phenotypes, based on their genotypes (Gaeta et al., 2024; Huang et al., 2024; Liu et al., 2024; Schuetz et al., 2024; Shen et al., 2024). However, it is challenging to predict the phenotype based on its genotypes, which are significantly different in variant distribution from the variants in training data with exact phenotype labels, because most of these models required similar variant distribution in protein or cDNA sequence. Additionally, genes are highly similar to time-series data, implying a high applicability of Recurrent Neural Network, like Long Short Term Mermory network (LSTM). However, a LSTM classifer was not learnable, without a descent in gradient, for the scoliosis high- and low-risk samples, probably due to the small sample number. Therefore, we transformed genotic information of *FBN1* sequences into a DCR space, with less discreteness. Thus, DCR-based models would be promising for such type of genotype-to-phenotype prediction.

## 5 Conclusion

In summary, scoliosis risk is predictable by deep learning based on genomic decomposed features of DCR. DCR-based classifier has predicted more scoliosis risk *FBN1* variants in ClinVar database. DCR-based models would be promising for genotype-to-phenotype prediction for more disease types.

## Data Availability

The datasets presented in this study can be found in online repositories. The names of the repository/repositories and accession number(s) can be found in the article/Supplementary Material.

## References

[B1] Al D. N. Wu N. Zhao S. Wu Z. Blank R. D. Zhang J. (2020). Kiaa1217: a novel candidate gene associated with isolated and syndromic vertebral malformations. Am. J. Med. Genet. A 182 (7), 1664–1672. 10.1002/ajmg.a.61607 32369272 PMC8128026

[B2] Bei-Guang N. Sen Z. Yu-Chang L. Xiao-Ping K. Yue-Hong C. Lin L. (2022). Convolutional neural networks based on sequential spike predict the high human adaptation of SARS-cov-2 omicron variants. Viruses 14 (5), 1–14. 10.3390/v14051072 PMC9147419 35632811

[B3] Buchan J. G. Alvarado D. M. Haller G. E. Cruchaga C. Harms M. B. Zhang T. (2014). Rare variants in fbn1 and fbn2 are associated with severe adolescent idiopathic scoliosis. Hum. Mol. Genet. 23 (19), 5271–5282. 10.1093/hmg/ddu224 24833718 PMC4159151

[B4] Cheng J. Novati G. Pan J. Bycroft C. Zemgulyte A. Applebaum T. (2023). Accurate proteome-wide missense variant effect prediction with alphamissense. Science. 381 (6664), eadg7492. 10.1126/science.adg7492 37733863

[B5] Cheng J. C. Castelein R. M. Chu W. C. Danielsson A. J. Dobbs M. B. Grivas T. B. (2015). Adolescent idiopathic scoliosis. Nat. Rev. Dis. Prim. 1, 15030. 10.1038/nrdp.2015.30 27188385

[B6] Choudhry M. N. Ahmad Z. Verma R. (2016). Adolescent idiopathic scoliosis. Open Orthop. J. 10, 143–154. 10.2174/1874325001610010143 27347243 PMC4897334

[B7] Cilli K. Tezeren G. Tas T. Bulut O. Ozturk H. Oztemur Z. (2009). School screening for scoliosis in sivas, Turkey. Acta Orthop. Traumatol. Turc. 43 (5), 426–430. 10.3944/AOTT.2009.426 19881324

[B8] Dofash L. Monahan G. V. Servian-Morilla E. Rivas E. Faiz F. Sullivan P. (2023). A klhl40 3' utr splice-altering variant causes milder nem8, an under-appreciated disease mechanism. Hum. Mol. Genet. 32 (7), 1127–1136. 10.1093/hmg/ddac272 36322148

[B9] Duance V. C. Crean J. K. Sims T. J. Avery N. Smith S. Menage J. (1998). Changes in collagen cross-linking in degenerative disc disease and scoliosis. SPINE 23 (23), 2545–2551. 10.1097/00007632-199812010-00009 9854753

[B10] Gaeta A. M. Quijada-Lopez M. Barbe F. Vaca R. Pujol M. Minguez O. (2024). Predicting alzheimer's disease csf core biomarkers: a multimodal machine learning approach. Front. Aging Neurosci. 16, 1369545. 10.3389/fnagi.2024.1369545 38988328 PMC11233742

[B11] Huang J. Osthushenrich T. MacNamara A. Malarstig A. Brocchetti S. Bradberry S. (2024). Proteomutametrics: machine learning approaches for solute carrier family 6 mutation pathogenicity prediction. RSC Adv. 14 (19), 13083–13094. 10.1039/d4ra00748d 38655474 PMC11034476

[B12] Jett K. Friedman J. M. (2010). Clinical and genetic aspects of neurofibromatosis 1. Genet. Med. 12 (1), 1–11. 10.1097/GIM.0b013e3181bf15e3 20027112

[B13] Jiang S. Zhang S. Kang X. Feng Y. Li Y. Nie M. (2023). Risk assessment of the possible intermediate host role of pigs for coronaviruses with a deep learning predictor. Viruses 15 (7), 1556. 10.3390/v15071556 37515242 PMC10384923

[B14] Jiang X. Liu F. Zhang M. Hu W. Zhao Y. Xia B. (2023). Advances in genetic factors of adolescent idiopathic scoliosis: a bibliometric analysis. Front. Pediatr. 11, 1301137. 10.3389/fped.2023.1301137 38322243 PMC10845672

[B15] Jo Y. Webster M. J. Kim S. Lee D. (2023). Interpretation of snp combination effects on schizophrenia etiology based on stepwise deep learning with multi-precision data. Brief. Funct. Genomics 23, 663–671. 10.1093/bfgp/elad041 PMC11428150 37738675

[B16] Jumper J. Evans R. Pritzel A. Green T. Figurnov M. Ronneberger O. (2021). Highly accurate protein structure prediction with alphafold. Nature 596 (7873), 583–589. 10.1038/s41586-021-03819-2 34265844 PMC8371605

[B17] Kikanloo S. R. Tarpada S. P. Cho W. (2019). Etiology of adolescent idiopathic scoliosis: a literature review. Asian Spine J. 13 (3), 519–526. 10.31616/asj.2018.0096 30744305 PMC6547389

[B18] Konieczny M. R. Senyurt H. Krauspe R. (2013). Epidemiology of adolescent idiopathic scoliosis. J. Child. Orthop. 7 (1), 3–9. 10.1007/s11832-012-0457-4 24432052 PMC3566258

[B19] Kotlarz K. Mielczarek M. Biecek P. Wojdak-Maksymiec K. Suchocki T. Topolski P. (2024). An explainable deep learning classifier of bovine mastitis based on whole-genome sequence data-circumventing the p >> n problem. Int. J. Mol. Sci. 25 (9), 4715. 10.3390/ijms25094715 38731932 PMC11083318

[B20] Kou I. Takahashi Y. Johnson T. A. Takahashi A. Guo L. Dai J. (2013). Genetic variants in gpr126 are associated with adolescent idiopathic scoliosis. Nat. Genet. 45 (6), 676–679. 10.1038/ng.2639 23666238

[B21] Kulis A. Gozdzialska A. Drag J. Jaskiewicz J. Knapik-Czajka M. Lipik E. (2015). Participation of sex hormones in multifactorial pathogenesis of adolescent idiopathic scoliosis. Int. Orthop. 39 (6), 1227–1236. 10.1007/s00264-015-2742-6 25804208

[B22] Landrum M. J. Lee J. M. Benson M. Brown G. Chao C. Chitipiralla S. (2016). Clinvar: public archive of interpretations of clinically relevant variants. Nucleic. acids. Res. 44 (D1), D862–D868. 10.1093/nar/gkv1222 26582918 PMC4702865

[B23] Landrum M. J. Lee J. M. Benson M. Brown G. R. Chao C. Chitipiralla S. (2018). Clinvar: improving access to variant interpretations and supporting evidence. Nucleic. acids. Res. 46 (D1), D1062–D1067. 10.1093/nar/gkx1153 29165669 PMC5753237

[B24] Landrum M. J. Lee J. M. Riley G. R. Jang W. Rubinstein W. S. Church D. M. (2014). Clinvar: public archive of relationships among sequence variation and human phenotype. Nucleic. acids. Res. 42 (Database issue), D980–D985. 10.1093/nar/gkt1113 24234437 PMC3965032

[B25] Li J. Tian F. Zhang S. Liu S. S. Kang X. P. Li Y. D. (2023). Genomic representation predicts an asymptotic host adaptation of bat coronaviruses using deep learning. Front. Microbiol. 14, 1157608. 10.3389/fmicb.2023.1157608 37213516 PMC10198438

[B26] Li J. Wu Y. N. Zhang S. Kang X. P. Jiang T. (2022). Deep learning based on biologically interpretable genome representation predicts two types of human adaptation of SARS-cov-2 variants. Brief. Bioinform. 23 (3), bbac036. 10.1093/bib/bbac036 35233612 PMC9116219

[B27] Li J. Zhang S. Li B. Hu Y. Kang X. Wu X. (2020). Machine learning methods for predicting human-adaptive influenza a viruses based on viral nucleotide compositions. Mol. Biol. Evol. 37 (4), 1224–1236. 10.1093/molbev/msz276 31750915 PMC7086167

[B28] Liu Y. Zhang T. You N. Wu S. Shen N. (2024). Magpie: accurate pathogenic prediction for multiple variant types using machine learning approach. Genome Med. 16 (1), 3. 10.1186/s13073-023-01274-4 38185709 PMC10773112

[B29] Minton K. (2023). Predicting variant pathogenicity with alphamissense. Nat. Rev. Genet. 24 (12), 804. 10.1038/s41576-023-00668-9 37821682

[B30] Ogura Y. Kou I. Miura S. Takahashi A. Xu L. Takeda K. (2015). A functional snp in bnc2 is associated with adolescent idiopathic scoliosis. Am. J. Hum. Genet. 97 (2), 337–342. 10.1016/j.ajhg.2015.06.012 26211971 PMC4573260

[B31] Otomo N. Lu H. F. Koido M. Kou I. Takeda K. Momozawa Y. (2021). Polygenic risk score of adolescent idiopathic scoliosis for potential clinical use. J. Bone. Min. Res. 36 (8), 1481–1491. 10.1002/jbmr.4324 34159637

[B32] Perez-Machado G. Berenguer-Pascual E. Bovea-Marco M. Rubio-Belmar P. A. Garcia-Lopez E. Garzon M. J. (2020). From genetics to epigenetics to unravel the etiology of adolescent idiopathic scoliosis. Bone 140, 115563. 10.1016/j.bone.2020.115563 32768685

[B33] Raczkowski J. W. (2007). The concentrations of testosterone and estradiol in girls with adolescent idiopathic scoliosis. Neuro Endocrinol. Lett. 28 (3), 302–304. 17627266

[B34] Schuetz R. J. Ceyhan D. Antoniou A. A. Chaudhari B. P. White P. (2024). Cnvoyant: a highly performant and explainable multi-classifier machine learning approach for determining the clinical significance of copy number variants. Res. Sq., 4308324. 10.21203/rs.3.rs-4308324/v1

[B35] Sharma S. Gao X. Londono D. Devroy S. E. Mauldin K. N. Frankel J. T. (2011). Genome-wide association studies of adolescent idiopathic scoliosis suggest candidate susceptibility genes. Hum. Mol. Genet. 20 (7), 1456–1466. 10.1093/hmg/ddq571 21216876 PMC3049353

[B36] Sharma S. Londono D. Eckalbar W. L. Gao X. Zhang D. Mauldin K. (2015). A pax1 enhancer locus is associated with susceptibility to idiopathic scoliosis in females. Nat. Commun. 6, 6452. 10.1038/ncomms7452 25784220 PMC4365504

[B37] Shen L. Falk M. J. Gai X. (2024). Mseqdr quick-mitome (qm): combining phenotype-guided variant interpretation and machine learning classifiers to aid primary mitochondrial disease genetic diagnosis. Curr. Protoc. 4 (1), e955. 10.1002/cpz1.955 38284225 PMC11046528

[B38] Sheng F. Xia C. Xu L. Qin X. Tang N. L. Qiu Y. (2019). New evidence supporting the role of fbn1 in the development of adolescent idiopathic scoliosis. SPINE 44 (4), E225–E232. 10.1097/BRS.0000000000002809 30044367

[B39] Soucacos P. N. Soucacos P. K. Zacharis K. C. Beris A. E. Xenakis T. A. (1997). School-screening for scoliosis. A prospective epidemiological study in northwestern and central Greece. J. Bone. Jt. Surg. Am. 79 (10), 1498–1503. 10.2106/00004623-199710000-00006 9378735

[B40] Stenson P. D. Ball E. V. Mort M. Phillips A. D. Shiel J. A. Thomas N. S. (2003). Human gene mutation database (hgmd): 2003 update. Hum. Mutat. 21 (6), 577–581. 10.1002/humu.10212 12754702

[B41] Sun W. Li J. Han P. Yang Y. Kang X. Li Y. (2014). U4 at the 3' utr of pb1 segment of h5n1 influenza virus promotes rna polymerase activity and contributes to viral pathogenicity. PLoS One. 9 (3), e93366. 10.1371/journal.pone.0093366 24676059 PMC3968160

[B42] Takahashi Y. Kou I. Takahashi A. Johnson T. A. Kono K. Kawakami N. (2011). A genome-wide association study identifies common variants near lbx1 associated with adolescent idiopathic scoliosis. Nat. Genet. 43 (12), 1237–1240. 10.1038/ng.974 22019779

[B43] Telenti A. Hodcroft E. B. Robertson D. L. (2022). The evolution and biology of SARS-cov-2 variants. Cold Spring Harb. Perspect. Med. 12 (5), a041390. 10.1101/cshperspect.a041390 35444005 PMC9159258

[B44] Ushiki A. Sheng R. R. Zhang Y. Zhao J. Nobuhara M. Murray E. (2024). Deletion of pax1 scoliosis-associated regulatory elements leads to a female-biased tail abnormality. Cell Rep. 43 (3), 113907. 10.1016/j.celrep.2024.113907 38461417 PMC11005513

[B45] Wong H. K. Hui J. H. Rajan U. Chia H. P. (2005). Idiopathic scoliosis in singapore schoolchildren: a prevalence study 15 years into the screening program. SPINE 30 (10), 1188–1196. 10.1097/01.brs.0000162280.95076.bb 15897834

[B46] Xi H. Peng Y. Xie W. Pang J. Ma N. Yang S. (2020). A chromosome 1q22 microdeletion including ash1l is associated with intellectual disability in a Chinese family. Mol. Cytogenet. 13, 20. 10.1186/s13039-020-00483-5 32518592 PMC7273683

[B47] Zhang S. Li Y. D. Cai Y. R. Kang X. P. Feng Y. Li Y. C. (2024). Compositional features analysis by machine learning in genome represents linear adaptation of monkeypox virus. Front. Genet. 15, 1361952. 10.3389/fgene.2024.1361952 38495668 PMC10940399

[B48] Zhu Z. Tang N. L. Xu L. Qin X. Mao S. Song Y. (2015). Genome-wide association study identifies new susceptibility loci for adolescent idiopathic scoliosis in Chinese girls. Nat. Commun. 6, 8355. 10.1038/ncomms9355 26394188 PMC4595747

