# Supplementary material for: Dinucleotide composition representation -based deep learning to predict scoliosis-associated Fibrillin-1 genotypes
Source: Front Genet. 2024 Oct 22;15:1492226. doi: 10.3389/fgene.2024.1492226 (PMC11534654; doi:10.3389/fgene.2024.1492226)
Supplement: Supplementary file 1 [file Table1.DOCX]

Supplementary Material

# Supplementary Figures and Tables

## Supplementary Table

**Supplementary Table 1.** High risk mutations for scoliosis predicted by DCR-based CNN predictor.

| Serial | label | mt $ | mt type | prediction | Score  _0 | Score  _1 | Annotation |
| --- | --- | --- | --- | --- | --- | --- | --- |
| 1862 | Pathogeniclikely |  | del  * | 1 | 0.07 | 0.93 | NM_000138.5(FBN1):c.8561del  (p.Leu2854fs) |
| 1863 | Pathogeniclikely |  | dup  ** | 1 | 0.02 | 0.98 | NM_000138.5(FBN1):c.8544dup  (p.Tyr2849fs) |
| 1865 | Pathogeniclikely |  | del | 1 | 0.02 | 0.98 | NM_000138.5(FBN1):c.8525_8529del (p.Leu2842fs) |
| 1868 | Pathogeniclikely |  | del | 1 | 0.01 | 0.99 | NM_000138.5(FBN1):c.8479del  (p.Tyr2827fs) |
| 1877 | Pathogeniclikely |  | del | 1 | 0.01 | 0.99 | NM_000138.5(FBN1):c.8329del  (p.Ile2777fs) |
| 1878 | Pathogeniclikely | C>T | SNP  *** | 1 | 0.31 | 0.69 | NM_000138.5(FBN1):c.8326C>T (p.Arg2776Ter) |
| 1879 | Pathogeniclikely |  | dup | 1 | 0.00 | 1.00 | NM_000138.5(FBN1):c.8252_8253dup (p.Ser2752Ter) |
| 1882 | Pathogeniclikely |  | del | 1 | 0.00 | 1.00 | NM_000138.5(FBN1):c.8203del  (p.Glu2735fs) |
| 1883 | Pathogeniclikely |  | del | 1 | 0.00 | 1.00 | NM_000138.5(FBN1):c.8176del  (p.Arg2726fs) |
| 1884 | Pathogeniclikely |  |  | 1 | 0.00 | 1.00 | NM_000138.5(FBN1):c.8148_8149insA (p.Glu2717fs) |
| 1886 | Pathogeniclikely |  | del | 1 | 0.00 | 1.00 | NM_000138.5(FBN1):c.8090_8099del (p.Pro2697fs) |
| 1887 | Pathogeniclikely |  | del | 1 | 0.00 | 1.00 | NM_000138.5(FBN1):c.8087del  (p.Asn2696fs) |
| 1906 | Pathogeniclikely |  | del_ins  **** | 1 | 0.00 | 1.00 | NM_000138.5(FBN1):c.7976_7977delinsTT (p.Cys2659Phe) |
| 1908 | Pathogeniclikely |  | del | 1 | 0.00 | 1.00 | NM_000138.5(FBN1):c.7931_7937del (p.Gly2644fs) |
| 1928 | Pathogeniclikely | G>A | SNP | 1 | 0.35 | 0.65 | NM_000138.5(FBN1):c.7806G>A (p.Trp2602Ter) |
| 1937 | Pathogeniclikely |  | del | 1 | 0.00 | 1.00 | NM_000138.5(FBN1):c.7735del  (p.His2579fs) |
| 1939 | Pathogeniclikely |  | del | 1 | 0.00 | 1.00 | NM_000138.5(FBN1):c.7719del  (p.Asn2574fs) |
| 1946 | Pathogeniclikely |  | del | 1 | 0.00 | 1.00 | NM_000138.5(FBN1):c.7678del  (p.Gln2560fs) |
| 1950 | Pathogeniclikely |  | del | 1 | 0.00 | 1.00 | NM_000138.5(FBN1):c.7643del  (p.Phe2548fs) |
| 1952 | Pathogeniclikely |  | dup | 1 | 0.00 | 1.00 | NM_000138.5(FBN1):c.7608dup  (p.Ser2537fs) |
| 1954 | Pathogeniclikely |  | del_ins | 1 | 0.00 | 1.00 | NM_000138.5(FBN1):c.7604_7605delinsTG (p.Cys2535Leu) |
| 1959 | Pathogeniclikely |  | del | 1 | 0.00 | 1.00 | NM_000138.5(FBN1):c.7577del  (p.Asn2526fs) |
| 1961 | Pathogeniclikely |  | del | 1 | 0.00 | 1.00 | NM_000138.5(FBN1):c.7574del  (p.Asn2525fs) |
| 1963 | Pathogeniclikely | C>A | SNP | 1 | 0.37 | 0.63 | NM_000138.5(FBN1):c.7566C>A (p.Cys2522Ter) |
| 1970 | Pathogeniclikely |  | del | 1 | 0.00 | 1.00 | NM_000138.5(FBN1):c.7497_7498del (p.Cys2500_Val2501insTer) |
| 1988 | Pathogeniclikely |  | del | 1 | 0.00 | 1.00 | NM_000138.5(FBN1):c.7399del  (p.Gln2467fs) |
| 1989 | Pathogeniclikely | G>T | SNP | 1 | 0.44 | 0.56 | NM_000138.5(FBN1):c.7387G>T (p.Glu2463Ter) |
| 2004 | Pathogeniclikely |  | del | 1 | 0.00 | 1.00 | NM_000138.5(FBN1):c.7259del  (p.Asn2420fs) |
| 2019 | Pathogeniclikely |  | dup | 1 | 0.00 | 1.00 | NM_000138.5(FBN1):c.7191dup  (p.Thr2398fs) |
| 2023 | Pathogeniclikely |  | del | 1 | 0.00 | 1.00 | NM_000138.5(FBN1):c.7151_7152del (p.Val2384fs) |
| 2025 | Pathogeniclikely |  | del | 1 | 0.00 | 1.00 | NM_000138.5(FBN1):c.7139del  (p.Phe2380fs) |
| 2027 | Pathogeniclikely |  | del | 1 | 0.00 | 1.00 | NM_000138.5(FBN1):c.7109del  (p.Gly2370fs) |
| 2033 | Pathogeniclikely |  | del | 1 | 0.00 | 1.00 | NM_000138.5(FBN1):c.7071del  (p.Val2358fs) |
| 2034 | Pathogeniclikely |  | del | 1 | 0.00 | 1.00 | NM_000138.5(FBN1):c.7022_7023del (p.Thr2341fs) |
| 2036 | Pathogeniclikely | C>G | SNP | 1 | 0.44 | 0.56 | NM_000138.5(FBN1):c.7014C>G (p.Tyr2338Ter) |
| 2040 | Pathogeniclikely |  | del_ins | 1 | 0.00 | 1.00 | NM_000138.5(FBN1):c.6964delinsGG (p.Phe2322fs) |
| 2041 | Pathogeniclikely | G>T | SNP | 1 | 0.46 | 0.54 | NM_000138.5(FBN1):c.6949G>T (p.Glu2317Ter) |
| 2042 | Pathogeniclikely |  | del | 1 | 0.00 | 1.00 | NM_000138.5(FBN1):c.6927del  (p.Asn2309fs) |
| 2052 | Pathogeniclikely | G>T | SNP | 1 | 0.44 | 0.56 | NM_000138.5(FBN1):c.6856G>T (p.Gly2286Ter) |
| 2060 | Pathogeniclikely |  | dup | 1 | 0.00 | 1.00 | NM_000138.5(FBN1):c.6793_6800dup (p.Leu2268fs) |
| 2062 | Pathogeniclikely |  | del | 1 | 0.00 | 1.00 | NM_000138.5(FBN1):c.6790del  (p.Glu2264fs) |
| 2064 | Pathogeniclikely |  | dup | 1 | 0.00 | 1.00 | NM_000138.5(FBN1):c.6770_6771dup (p.Cys2258fs) |
| 2076 | Pathogeniclikely |  | del | 1 | 0.00 | 1.00 | NM_000138.5(FBN1):c.6705del  (p.Tyr2236fs) |
| 2077 | Pathogeniclikely |  | del | 1 | 0.00 | 1.00 | NM_000138.5(FBN1):c.6704del  (p.Gly2235fs) |
| 2086 | Pathogeniclikely |  | dup | 1 | 0.00 | 1.00 | NM_000138.5(FBN1):c.6670dup  (p.Thr2224fs) |
| 2088 | Pathogeniclikely |  | dup | 1 | 0.00 | 1.00 | NM_000138.5(FBN1):c.6656dup  (p.Arg2220fs) |
| 2112 | Pathogeniclikely |  | dup | 1 | 0.00 | 1.00 | NM_000138.5(FBN1):c.6553_6556dup (p.Gly2186fs) |
| 2115 | Pathogeniclikely |  | del | 1 | 0.00 | 1.00 | NM_000138.5(FBN1):c.6524del  (p.Pro2175fs) |
| 2145 | Pathogeniclikely | G>T | SNP | 1 | 0.38 | 0.62 | NM_000138.5(FBN1):c.6340G>T (p.Gly2114Ter) |
| 2151 | Pathogeniclikely | G>A | SNP | 1 | 0.44 | 0.56 | NM_000138.5(FBN1):c.6276G>A (p.Trp2092Ter) |
| 2173 | Pathogeniclikely |  | del | 1 | 0.00 | 1.00 | NM_000138.5(FBN1):c.6080del  (p.Gly2027fs) |
| 2176 | Pathogeniclikely |  | dup | 1 | 0.00 | 1.00 | NM_000138.5(FBN1):c.6070dup  (p.Cys2024fs) |
| 2184 | Pathogeniclikely | C>T | SNP | 1 | 0.49 | 0.51 | NM_000138.5(FBN1):c.6019C>T (p.Gln2007Ter) |
| 2185 | Pathogeniclikely |  |  | 1 | 0.00 | 1.00 | NM_000138.5(FBN1):c.6013_6014insTA (p.Ser2005fs) |
| 2186 | Pathogeniclikely |  | del | 1 | 0.00 | 1.00 | NM_000138.5(FBN1):c.6012_6013del (p.Tyr2004_Ser2005delinsTer) |
| 2187 | Pathogeniclikely |  | dup | 1 | 0.00 | 1.00 | NM_000138.5(FBN1):c.6006_6012dup (p.Ser2005fs) |
| 2200 | Pathogeniclikely |  | del | 1 | 0.00 | 1.00 | NM_000138.5(FBN1):c.5943del  (p.Arg1982fs) |
| 2203 | Pathogeniclikely |  | dup | 1 | 0.00 | 1.00 | NM_000138.5(FBN1):c.5919dup  (p.Ile1974fs) |
| 2210 | Pathogeniclikely |  | del | 1 | 0.00 | 1.00 | NM_000138.5(FBN1):c.5888del  (p.Glu1963fs) |
| 2213 | Pathogeniclikely | C>A | SNP | 1 | 0.35 | 0.65 | NM_000138.5(FBN1):c.5874C>A (p.Cys1958Ter) |
| 2215 | Pathogeniclikely | C>T | SNP | 1 | 0.46 | 0.54 | NM_000138.5(FBN1):c.5869C>T (p.Gln1957Ter) |
| 2245 | Pathogeniclikely |  |  | 1 | 0.00 | 1.00 | NM_000138.5(FBN1):c.5667_5668insTTGGA (p.Ile1892fs) |
| 2254 | Pathogeniclikely | C>T | SNP | 1 | 0.44 | 0.56 | NM_000138.5(FBN1):c.5560C>T (p.Gln1854Ter) |
| 2267 | Pathogeniclikely |  | dup | 1 | 0.00 | 1.00 | NM_000138.5(FBN1):c.5499dup  (p.Asp1834Ter) |
| 2291 | Pathogeniclikely | T>A | SNP | 1 | 0.49 | 0.51 | NM_000138.5(FBN1):c.5280T>A (p.Tyr1760Ter) |
| 2295 | Pathogeniclikely | C>T | SNP | 1 | 0.49 | 0.51 | NM_000138.5(FBN1):c.5251C>T (p.Gln1751Ter) |
| 2300 | Pathogeniclikely |  | del | 1 | 0.00 | 1.00 | NM_000138.5(FBN1):c.5179del  (p.Arg1727fs) |
| 2305 | Pathogeniclikely |  | del | 1 | 0.00 | 1.00 | NM_000138.5(FBN1):c.5066del  (p.Asp1689fs) |
| 2311 | Pathogeniclikely |  | dup | 1 | 0.00 | 1.00 | NM_000138.5(FBN1):c.5016dup  (p.Ile1673fs) |
| 2321 | Pathogeniclikely | C>T | SNP | 1 | 0.44 | 0.56 | NM_000138.5(FBN1):c.4930C>T (p.Arg1644Ter) |
| 2340 | Pathogeniclikely |  | del | 1 | 0.00 | 1.00 | NM_000138.5(FBN1):c.4700del  (p.Gly1567fs) |
| 2345 | Pathogeniclikely |  | del | 1 | 0.00 | 1.00 | NM_000138.5(FBN1):c.4649del  (p.Ser1550fs) |
| 2346 | Pathogeniclikely |  | del | 1 | 0.00 | 1.00 | NM_000138.5(FBN1):c.4629del  (p.Asp1543fs) |
| 2354 | Pathogeniclikely |  | del | 1 | 0.00 | 1.00 | NM_000138.5(FBN1):c.4579_4580del (p.Cys1526_Val1527insTer) |
| 2356 | Pathogeniclikely | C>T | SNP | 1 | 0.37 | 0.63 | NM_000138.5(FBN1):c.4567C>T (p.Arg1523Ter) |
| 2375 | Pathogeniclikely |  | del | 1 | 0.00 | 1.00 | NM_000138.5(FBN1):c.4474del  (p.Leu1492fs) |
| 2384 | Pathogeniclikely |  | dup | 1 | 0.00 | 1.00 | NM_000138.5(FBN1):c.4407_4409dup (p.Cys1470dup) |
| 2390 | Pathogeniclikely |  | dup | 1 | 0.00 | 1.00 | NM_000138.5(FBN1):c.4346_4349dup (p.Cys1450Ter) |
| 2398 | Pathogeniclikely |  | del | 1 | 0.00 | 1.00 | NM_000138.5(FBN1):c.4311del  (p.Ser1438fs) |
| 2399 | Pathogeniclikely |  |  | 1 | 0.00 | 1.00 | NM_000138.5(FBN1):c.4301_4302insAG (p.Phe1435fs) |
| 2400 | Pathogeniclikely |  | del | 1 | 0.00 | 1.00 | NM_000138.5(FBN1):c.4301del  (p.Gly1434fs) |
| 2402 | Pathogeniclikely |  | del | 1 | 0.00 | 1.00 | NM_000138.5(FBN1):c.4287_4288del (p.Cys1429_Glu1430delinsTer) |
| 2409 | Pathogeniclikely |  | del | 1 | 0.00 | 1.00 | NM_000138.5(FBN1):c.4226_4227del (p.Cys1408_Ser1409insTer) |
| 2414 | Pathogeniclikely |  |  | 1 | 0.00 | 1.00 | NM_000138.5(FBN1):c.4196_4197insA (p.Phe1400fs) |
| 2415 | Pathogeniclikely |  | del | 1 | 0.00 | 1.00 | NM_000138.5(FBN1):c.4197del  (p.Phe1400fs) |
| 2427 | Pathogeniclikely |  | del | 1 | 0.00 | 1.00 | NM_000138.5(FBN1):c.4056del  (p.Trp1354fs) |
| 2432 | Pathogeniclikely |  | del | 1 | 0.00 | 1.00 | NM_000138.5(FBN1):c.4032_4033del (p.Ser1345fs) |
| 2435 | Pathogeniclikely |  | del | 1 | 0.00 | 1.00 | NM_000138.5(FBN1):c.4007_4010del (p.His1336fs) |
| 2436 | Pathogeniclikely |  | del | 1 | 0.00 | 1.00 | NM_000138.5(FBN1):c.3993del  (p.His1331fs) |
| 2444 | Pathogeniclikely |  | del | 1 | 0.00 | 1.00 | NM_000138.5(FBN1):c.3945del  (p.Gly1316fs) |
| 2451 | Pathogeniclikely |  | dup | 1 | 0.00 | 1.00 | NM_000138.5(FBN1):c.3893dup  (p.Asn1298fs) |
| 2461 | Pathogeniclikely |  | del | 1 | 0.00 | 1.00 | NM_000138.5(FBN1):c.3783del (p.Glu1260_Tyr1261insTer) |
| 2462 | Pathogeniclikely |  | del | 1 | 0.00 | 1.00 | NM_000138.5(FBN1):c.3759del  (p.Gln1253fs) |
| 2471 | Pathogeniclikely |  | del | 1 | 0.00 | 1.00 | NM_000138.5(FBN1):c.3640_3641del (p.Asn1214fs) |
| 2498 | Pathogeniclikely |  | dup | 1 | 0.00 | 1.00 | NM_000138.5(FBN1):c.3487_3488dup (p.Leu1165fs) |
| 2501 | Pathogeniclikely |  | del_ins | 1 | 0.00 | 1.00 | NM_000138.4(FBN1):c.3465_3466delinsAAG (p.Asp1155fs) |
| 2507 | Pathogeniclikely |  | del | 1 | 0.00 | 1.00 | NM_000138.5(FBN1):c.3444del  (p.Asn1149fs) |
| 2515 | Pathogeniclikely | C>T | SNP | 1 | 0.43 | 0.57 | NM_000138.5(FBN1):c.3373C>T (p.Arg1125Ter) |
| 2528 | Pathogeniclikely |  | dup | 1 | 0.00 | 1.00 | NM_000138.5(FBN1):c.3320_3323dup (p.Met1108fs) |
| 2533 | Pathogeniclikely |  | del | 1 | 0.00 | 1.00 | NM_000138.5(FBN1):c.3274del  (p.Asp1092fs) |
| 2544 | Pathogeniclikely |  | del | 1 | 0.00 | 1.00 | NM_000138.5(FBN1):c.3193del  (p.Glu1065fs) |
| 2546 | Pathogeniclikely | T>A | SNP | 1 | 0.38 | 0.62 | NM_000138.5(FBN1):c.3165T>A (p.Cys1055Ter) |
| 2551 | Pathogeniclikely |  | del | 1 | 0.00 | 1.00 | NM_000138.5(FBN1):c.3140_3141del (p.Thr1047fs) |
| 2552 | Pathogeniclikely |  | del | 1 | 0.00 | 1.00 | NM_000138.5(FBN1):c.3126del  (p.Lys1043fs) |
| 2557 | Pathogeniclikely | G>T | SNP | 1 | 0.38 | 0.62 | NM_000138.5(FBN1):c.3064G>T (p.Gly1022Ter) |
| 2560 | Pathogeniclikely | G>T | SNP | 1 | 0.46 | 0.54 | NM_000138.5(FBN1):c.3007G>T (p.Glu1003Ter) |
| 2563 | Pathogeniclikely |  | del | 1 | 0.00 | 1.00 | NM_000138.5(FBN1):c.2948_2970del (p.Cys982_Ser983insTer) |
| 2571 | Pathogeniclikely |  | dup | 1 | 0.00 | 1.00 | NM_000138.5(FBN1):c.2934dup  (p.Ala979fs) |
| 2573 | Pathogeniclikely |  | del | 1 | 0.00 | 1.00 | NM_000138.5(FBN1):c.2913del  (p.Ile971fs) |
| 2574 | Pathogeniclikely |  | del | 1 | 0.00 | 1.00 | NM_000138.5(FBN1):c.2884_2890del (p.Tyr962fs) |
| 2580 | Pathogeniclikely |  | del | 1 | 0.00 | 1.00 | NM_000138.5(FBN1):c.2833del  (p.Ala945fs) |
| 2581 | Pathogeniclikely |  | del | 1 | 0.00 | 1.00 | NM_000138.5(FBN1):c.2830del  (p.Asp944fs) |
| 2584 | Pathogeniclikely |  | del | 1 | 0.00 | 1.00 | NM_000138.5(FBN1):c.2764_2780del (p.Cys921_Lys922insTer) |
| 2595 | Pathogeniclikely |  | del | 1 | 0.00 | 1.00 | NM_000138.5(FBN1):c.2714del  (p.Gly905fs) |
| 2596 | Pathogeniclikely |  |  | 1 | 0.00 | 1.00 | NM_000138.5(FBN1):c.2698_2699insGG (p.Tyr900fs) |
| 2599 | Pathogeniclikely |  | dup | 1 | 0.00 | 1.00 | NM_000138.5(FBN1):c.2691dup  (p.Lys898Ter) |
| 2601 | Pathogeniclikely |  | del | 1 | 0.00 | 1.00 | NM_000138.5(FBN1):c.2682del  (p.Ile895fs) |
| 2614 | Pathogeniclikely |  | del | 1 | 0.00 | 1.00 | NM_000138.5(FBN1):c.2631del  (p.Ser878fs) |
| 2620 | Pathogeniclikely | C>T | SNP | 1 | 0.45 | 0.55 | NM_000138.5(FBN1):c.2563C>T  (p.Gln855Ter) |
| 2626 | Pathogeniclikely |  | del | 1 | 0.00 | 1.00 | NM_000138.5(FBN1):c.2529del  (p.Ile844fs) |
| 2633 | Pathogeniclikely |  | del | 1 | 0.00 | 1.00 | NM_000138.5(FBN1):c.2474del  (p.Pro825fs) |
| 2634 | Pathogeniclikely |  |  | 1 | 0.00 | 1.00 | NM_000138.5(FBN1):c.2467_2468insG (p.Asn823fs) |
| 2646 | Pathogeniclikely |  | dup | 1 | 0.00 | 1.00 | NM_000138.5(FBN1):c.2364dup  (p.Val789fs) |
| 2648 | Pathogeniclikely |  | del | 1 | 0.00 | 1.00 | NM_000138.5(FBN1):c.2342del  (p.Cys781fs) |
| 2668 | Pathogeniclikely |  | del | 1 | 0.00 | 1.00 | NM_000138.5(FBN1):c.2186del  (p.Leu729fs) |
| 2670 | Pathogeniclikely |  | del | 1 | 0.00 | 1.00 | NM_000138.5(FBN1):c.2176del  (p.Glu726fs) |
| 2674 | Pathogeniclikely |  | del | 1 | 0.00 | 1.00 | NM_000138.5(FBN1):c.2118_2127del (p.Glu706fs) |
| 2685 | Pathogeniclikely |  | del | 1 | 0.00 | 1.00 | NM_000138.5(FBN1):c.2034_2052del (p.Thr679fs) |
| 2691 | Pathogeniclikely |  | del_ins | 1 | 0.00 | 1.00 | NM_000138.5(FBN1):c.1962_1981delinsAG (p.Asp654_Cys661delinsGluGly) |
| 2695 | Pathogeniclikely |  | dup | 1 | 0.00 | 1.00 | NM_000138.5(FBN1):c.1948dup  (p.Arg650fs) |
| 2697 | Pathogeniclikely |  | del_ins | 1 | 0.00 | 1.00 | NM_000138.5(FBN1):c.1916_1917delinsTG (p.Cys639Leu) |
| 2714 | Pathogeniclikely | C>A | SNP | 1 | 0.45 | 0.55 | NM_000138.5(FBN1):c.1817C>A  (p.Ser606Ter) |
| 2722 | Pathogeniclikely |  | del | 1 | 0.00 | 1.00 | NM_000138.5(FBN1):c.1744_1745del (p.Cys582fs) |
| 2723 | Pathogeniclikely |  | del | 1 | 0.00 | 1.00 | NM_000138.5(FBN1):c.1737del  (p.Asn580fs) |
| 2728 | Pathogeniclikely |  | del | 1 | 0.00 | 1.00 | NM_000138.5(FBN1):c.1709del  (p.Cys570fs) |
| 2731 | Pathogeniclikely |  | dup | 1 | 0.00 | 1.00 | NM_000138.5(FBN1):c.1669_1677dup (p.Cys557_Ala559dup) |
| 2735 | Pathogeniclikely | C>A | SNP | 1 | 0.43 | 0.57 | NM_000138.5(FBN1):c.1665C>A  (p.Cys555Ter) |
| 2741 | Pathogeniclikely |  | del | 1 | 0.00 | 1.00 | NM_000138.5(FBN1):c.1617del  (p.Ile540fs) |
| 2747 | Pathogeniclikely |  | del | 1 | 0.00 | 1.00 | NM_000138.5(FBN1):c.1561_1562del (p.Ser521fs) |
| 2753 | Pathogeniclikely |  | del | 1 | 0.00 | 1.00 | NM_000138.5(FBN1):c.1520del  (p.Asn507fs) |
| 2770 | Pathogeniclikely | C>T | SNP | 1 | 0.47 | 0.53 | NM_000138.5(FBN1):c.1285C>T  (p.Arg429Ter) |
| 2771 | Pathogeniclikely |  | del | 1 | 0.00 | 1.00 | NM_000138.5(FBN1):c.1211del  (p.Pro404fs) |
| 2772 | Pathogeniclikely |  | dup | 1 | 0.00 | 1.00 | NM_000138.5(FBN1):c.1185dup  (p.Pro396fs) |
| 2775 | Pathogeniclikely |  | del | 1 | 0.00 | 1.00 | NM_000138.5(FBN1):c.1160del  (p.Lys387fs) |
| 2782 | Pathogeniclikely |  | del | 1 | 0.00 | 1.00 | NM_000138.5(FBN1):c.1117del  (p.Ala373fs) |
| 2797 | Pathogeniclikely |  | del | 1 | 0.00 | 1.00 | NM_000138.5(FBN1):c.961_962del  (p.Thr321fs) |
| 2798 | Pathogeniclikely |  | dup | 1 | 0.00 | 1.00 | NM_000138.5(FBN1):c.959dup  (p.Tyr320Ter) |
| 2804 | Pathogeniclikely |  | del | 1 | 0.00 | 1.00 | NM_000138.5(FBN1):c.799_805del  (p.Gly267fs) |
| 2808 | Pathogeniclikely | C>T | SNP | 1 | 0.44 | 0.56 | NM_000138.5(FBN1):c.733C>T  (p.Gln245Ter) |
| 2819 | Pathogeniclikely |  | del | 1 | 0.00 | 1.00 | NM_000138.5(FBN1):c.590del  (p.Gly197fs) |
| 2820 | Pathogeniclikely |  | del | 1 | 0.00 | 1.00 | NM_000138.5(FBN1):c.561del  (p.Phe187fs) |
| 2824 | Pathogeniclikely |  | dup | 1 | 0.00 | 1.00 | NM_000138.5(FBN1):c.531dup  (p.Glu178Ter) |
| 2827 | Pathogeniclikely |  | del | 1 | 0.00 | 1.00 | NM_000138.5(FBN1):c.510del (p.Thr169_Tyr170insTer) |
| 2838 | Pathogeniclikely |  | del | 1 | 0.00 | 1.00 | NM_000138.5(FBN1):c.488del  (p.Pro163fs) |
| 2844 | Pathogeniclikely |  | del | 1 | 0.00 | 1.00 | NM_000138.5(FBN1):c.458del  (p.Gly153fs) |
| 2849 | Pathogeniclikely |  | del | 1 | 0.00 | 1.00 | NM_000138.5(FBN1):c.441del  (p.Gln147fs) |
| 2856 | Pathogeniclikely |  | del | 1 | 0.00 | 1.00 | NM_000138.5(FBN1):c.390del  (p.Ser130fs) |
| 2863 | Pathogeniclikely |  | del | 1 | 0.00 | 1.00 | NM_000138.5(FBN1):c.320_321del  (p.Ile107fs) |
| 2881 | Pathogeniclikely |  | dup | 1 | 0.00 | 1.00 | NM_000138.5(FBN1):c.201_203dup (p.Cys68dup) |
| 2894 | Pathogeniclikely |  | del | 1 | 0.00 | 1.00 | NM_000138.5(FBN1):c.51_52del  (p.Leu17fs) |
| 2895 | Pathogeniclikely |  | del | 1 | 0.00 | 1.00 | NM_000138.5(FBN1):c.52del  (p.Ala18fs) |
| 2896 | Pathogeniclikely |  | del | 1 | 0.00 | 1.00 | NM_000138.5(FBN1):c.32_42del  (p.Leu11fs) |
| 9649 | Uncertain |  | del | 1 | 0.07 | 0.93 | NM_000138.5(FBN1):c.8562del  (p.Ser2855fs) |
| 9679 | Uncertain |  | del | 1 | 0.01 | 0.99 | NM_000138.5(FBN1):c.8428del  (p.Glu2810fs) |
| 9687 | Uncertain |  | del | 1 | 0.01 | 0.99 | NM_000138.5(FBN1):c.8396del  (p.Asn2799fs) |
| 9701 | Uncertain |  | dup | 1 | 0.00 | 1.00 | NM_000138.5(FBN1):c.8312dup  (p.Ser2772fs) |
| 9791 | Uncertain |  | dup | 1 | 0.00 | 1.00 | NM_000138.5(FBN1):c.7735_7746dup (p.His2579_Gln2582dup) |
| 10076 | Uncertain |  | dup | 1 | 0.00 | 1.00 | NM_000138.5(FBN1):c.6038_6040dup (p.Asp2013_Ile2014insAsn) |
| 10092 | Uncertain |  | del_ins | 1 | 0.00 | 1.00 | NM_000138.5(FBN1):c.5980_5981delinsTT (p.Gly1994Leu) |
| 10139 | Uncertain |  | dup | 1 | 0.00 | 1.00 | NM_000138.5(FBN1):c.5746_5748dup (p.Cys1916dup) |
| 10225 | Uncertain |  | dup | 1 | 0.00 | 1.00 | NM_000138.5(FBN1):c.5258_5260dup (p.Pro1753_Gly1754insAla) |
| 10242 | Uncertain |  | del_ins | 1 | 0.00 | 1.00 | NM_000138.5(FBN1):c.5101_5102delinsTT (p.Ala1701Phe) |
| 10462 | Uncertain |  | dup | 1 | 0.00 | 1.00 | NM_000138.5(FBN1):c.3625_3627dup (p.Glu1209_Thr1210insGlu) |
| 10466 | Uncertain |  | del_ins | 1 | 0.00 | 1.00 | NM_000138.5(FBN1):c.3605_3606delinsTT (p.Ser1202Ile) |
| 10479 | Uncertain |  | del_ins | 1 | 0.00 | 1.00 | NM_000138.5(FBN1):c.3569_3570delinsAA (p.Pro1190Gln) |
| 10525 | Uncertain |  | del_ins | 1 | 0.00 | 1.00 | NM_000138.4(FBN1):c.3232_3236delinsATCTC (p.Pro1078_Asp1079delinsIleSer) |
| 10572 | Uncertain |  | del_ins | 1 | 0.00 | 1.00 | NM_000138.5(FBN1):c.2964_2965delinsTT (p.Trp988_Gly989delinsCysCys) |
| 10760 | Uncertain |  | del_ins | 1 | 0.00 | 1.00 | NM_000138.5(FBN1):c.1929_1930delinsTT (p.Ala644Ser) |
| 11081 | Uncertain |  | del_ins | 1 | 0.00 | 1.00 | NM_000138.5(FBN1):c.155_156delinsTT (p.Ala52Val) |

$mt: mutation, * del: deletion; ** dup: duplication; *** SNP: single nucleotide polymorphism; **** del_ins: deletion and insertion.
